# Music Teachers’ Perspectives and Experiences of Ensemble and Learning Skills

**DOI:** 10.3389/fpsyg.2020.00291

**Published:** 2020-03-06

**Authors:** Andrea Schiavio, Mats B. Küssner, Aaron Williamon

**Affiliations:** ^1^Centre for Systematic Musicology, University of Graz, Graz, Austria; ^2^Institut für Musikwissenschaft und Medienwissenschaft, Humboldt-Universität zu Berlin, Berlin, Germany; ^3^Centre for Performance Science, Royal College of Music, London, United Kingdom; ^4^Faculty of Medicine, Imperial College London, London, United Kingdom

**Keywords:** musical learning, music teacher, ensemble skills, learning skills, instrumental music education

## Abstract

In this article, we report data from two survey studies administered to expert music teachers. Both questionnaires aimed to explore teachers’ pedagogical and performative practice and included open questions elucidating musical skills emerging in groups. The first study focuses on collective teaching settings offered to amateurs, jazz musicians, and university students with various levels of musical expertise. The second reports data from teachers based at the Royal College of Music, London, where the main emphasis is on Western classical repertoire. We integrate both studies and discuss overlapping findings. Despite intrinsic differences concerning the general goals of their teaching and the educational systems in which they operate, our data indicate the ability to “listen and respond to others” as the most important ensemble skill, whereas “time management,” “comparing yourself to the class,” and the “development of responsible ways of learning” emerged as main learning skills. We discuss results and suggestions for future research in teaching and learning music in different contexts in the light of recent theoretical research in the cognitive sciences, considering implications for educators interested in diverse skill levels.

## Introduction

In Western classical musical contexts, the process of learning to play an instrument is often framed within a combination of individual practice and one-to-one lessons with a music teacher (Creech and Gaunt, 2018). This master-apprentice model has a long tradition and continues to play an important role in current pedagogical systems in music, and beyond. As reported by Calvert (2014), learning based on craft apprenticeship remains a fundamental aspect of contemporary education, and it receives growing attention in research concerning a rich variety of domains, ranging from government-related careers (Fuller and Unwin, 2007) to the arts (Nerland and Hanken, 2004). Defined as “an agreement between a skilled person and an unskilled person, whereby the unskilled person learns to practice a specialized craft” (Coy, 1989, p. 3), such a learning format is usually implemented in musical settings through adaptable rules negotiated by a learner and an educator. Ideally, this can facilitate skill development in the former and help the latter monitor the student’s progress over time (see Sosniak, 1990). Consider, for example, how a classical guitar student can contribute to designing a curriculum with his or her teacher where the planned repertoire involves flamenco, jazz, or folk music, and where transcriptions from non-classical literature could become the main focus of the student’s learning trajectory and not just a deviation from the standard set of pieces to be studied. Not only can similar forms of close collaboration stimulate the learner in taking a more active role in the dynamics of, and preparation for, the class, they can also help the teacher better address critical weaknesses of the student (e.g., control of each voice in a polyphonic piece, accuracy in tremolo technique, etc.) and provide a useful meeting point for discussion, constructive criticism, and mutual reflection (see Elliott and Silverman, 2015; van der Schyff et al., 2016; Schiavio, 2019).

There is a growing body of work that examines the interactive dynamics between students and their teachers in musical settings (see Siebenaler, 1997; Rostvall and West, 2003; Zhukov, 2012). This research offers a rich overview of the many positive outcomes of one-to-one pedagogical settings (see Davidson and Jordan, 2007; Creech, 2012). Nonetheless, while individual tuition can indeed lead to a number of optimal learning experiences, and is flexible enough to be adapted to the various needs of students and teachers (Gaunt, 2011), it is also necessarily limited. As reported by Collens and Creech (2013), one major weakness is that “one-to-one tuition can develop into a site of interpersonal conflict and high anxiety where the relationship itself can become an obstruction to learning” (p. 151). Along these lines, Almeida de Sa Serra Dawa (2010) showed that 40% of 64 music students who took part in a study, wished to change teacher in light of interpersonal issues (reported in Daniel and Parkes, 2015, p. 111). Indeed, problems based on hierarchy and control are not so uncommon in one-to-one teaching settings: recently, Daniel and Parkes (2015) reviewed research that addresses this problem in detail and noted how issues of power can be dangerous for a positive learning experience, leading to negative outcomes and psychological harm (Creech and Hallam, 2011; Gaunt, 2011).

With this in mind, recent theory and research in music education is concerned with forms of learning that complement traditional one-to-one settings in various ways. One line of enquiry of particular interest for this paper is focused on the properties and possibilities offered by collaborative forms of music making (see Luce, 2001; Nielsen et al., 2018). In the present contribution, we aim to investigate how music teachers working in different contexts engage in such collective practices. Within this area, much work is focused on the ability of students to assess their peers (e.g., Lebler, 2008; see also Searby and Ewers, 1997), participate actively in the unfolding dynamics of the lesson (e.g., Schiavio et al., 2018), and take up responsibilities for their own learning and personal growth (e.g., O’Neill, 2010; van der Schyff, 2019). Such settings are found highly beneficial for improving social cohesion, empathy, creativity, and wellbeing among other aspects (Burnard et al., 2008; Gaunt and Westerlund, 2013).

In this article, we contribute to this line of research by exploring music teachers’ experiences and perceptions of (i) *Ensemble skills* and (ii) *Learning skills*. The former refers to the interpersonal ability to foster and maintain good social and performative dynamics within groups of learners involved in joint music-making. The latter defines the general set of abilities supporting musical skill acquisition. Given that communication and control issues were flagged as possible risks in reports examining individual tuition, it is important to get novel insights into just how these aspects are addressed and perceived in collaborative and performative settings. This can, in turn, offer advantages for research and practice in musical learning. Understanding what kind of learning skills are enhanced within a safe social environment can help teachers compensate for the potential loss of individually tailored moments in collective tuition, and better account for the diverse ways in which students can reciprocally interact and flourish. We discuss such insights through the lens of the “embodied” approach to mind, a perspective from the cognitive sciences which emphasizes the key role of first-hand experience and (inter)action for learning, performing, and achieving cognitive tasks more generally (see Varela et al., 1991; Thompson, 2007). Before exploring this framework in more detail, we first report and integrate qualitative data from two different studies with music teachers dealing with students of varying expertise levels. The first study focuses on music educators who teach (or have been teaching) amateurs, jazz musicians, children, and university-level students in both individual and collective contexts. These include pedagogical settings based on private lessons, popular music academies, conservatoires, and jazz schools in North America and Europe. This vast range of backgrounds brought forth descriptions of teaching experiences involving students with different goals, motivations, and expectations. In the second study, by contrast, all participants are teachers working at the Royal College of Music, London, where the main emphasis is on Western classical performance and composition. Importantly, while data on *Ensemble skills* refer in most cases to collective forms of music making, the reported experiences of *Learning skills* involve a focus on both individual and collective settings. Bringing together both studies can help offer insights that go beyond collaborative musical learning occurring in specific settings only (e.g., with expert students). Having presented the empirical work, we discuss overlapping findings and conclude with suggestions for future research and theory concerning collaborative musical learning.

## Study 1

### Methods

#### Participants

A total of 14 instrumental music teachers (10 women) working in different pedagogical settings took part in the study (age: *M* = 41.5 years, range: 27–59 years). Data from 11 participants were analyzed and discussed in another article (Schiavio et al., 2018) but were re-analyzed for the present contribution. While the teachers came from diverse cultural and geographical backgrounds, they were all required to have broad familiarity with individual and collective pedagogical settings, as well as experience as performers. In particular, a minimum of 10 years’ experience studying their instrument was required. Most of them were pianists (*n* = 8), while others taught guitar (*n* = 2), drums (*n* = 1), flute (*n* = 1), violin (*n* = 1), and organ (*n* = 1). Many of the respondents specialized in classical music (*n* = 10), followed by jazz (*n* = 2), improvisation (*n* = 1), and experimental music (*n* = 1). Ethical approval for data collection was granted by the Research Ethics Committee of the University of Graz, and all participants provided written informed consent before taking part in the study.

#### Materials and Procedure

Most participants (*n* = 8) received an open-ended survey to complete electronically (e.g., via computer) and were instructed to return it by email when completed. Following an initial section where information on musical background and demography was collected, the survey consisted of 18 items in total, to which participants were asked to respond freely and without a word limit. The survey was organized into two main sections: the first (questions 1–11) elicited responses on the teachers’ experience of one-to-one settings. Here, open-ended items included: “please describe how you prepare for your lessons as a teacher or student” and “what are the aspects that you feel contribute most to the development of your skills?” The second section (questions 12–18) focused on themes associated with collective tuition. Examples included: “what kind of corporeal sensations do you feel during collective lessons? On what do they depend? How could these be improved?” The remaining participants (*n* = 6) were interviewed individually, following the themes and structure of the survey. All interviews were audio recorded and were conducted either in English or Italian. Data relevant for the current study (i.e., responses that fit the two pre-determined categories reported below) were then transcribed verbatim. The duration of interviews ranged from 25 to 33 min.

#### Data Analysis

Data from the survey and interviews were analyzed following a deductive thematic analysis based on two pre-determined categories: *Ensemble skills* and *Learning skills*. The analysis involved four main steps: familiarization, categorization, interpretation, and integration. The first step (familiarization) involved an immersion in the raw dataset, which aimed to achieve familiarity with the range of themes emerging from both written responses and audio-recordings. The second phase (categorization) started with the selection and segmentation of all relevant material, followed by a classification of the data per code – namely, data could either fall into the category of *Ensemble skills* or *Learning skills*. Here, we selected material from nine participants, whose quotes were deemed most appropriate for these categories. In the third step (interpretation), all selected excerpts were verified by the research team and then re-organized if needed. In the final step (integration), data were discussed and compared with the results of study 2 to provide an integrated view of the topics being covered.

#### Results

##### Ensemble skills

A way to explore the complex phenomenology of collaborative musical learning involves identifying those music-related skills that develop collectively and that serve a beneficial function to improve the pedagogical setting from which they emerge. To do so, however, we first need to clarify what really differentiates collective learning from master-apprentice models. Consider the following two short quotes from a female guitarist:

“I really like seeing my students create spontaneously, and enjoy, brief moments of musical freedom.”

“Sometimes they [my students] just play together and other times they exchange mutual worries about certain aspects of the piece.”

Playing and learning together involves more than simply repeating what is on the score, or facing a particular problem. It is an experience that also encourages open dialogues (i.e., with peers), as well as free musical explorations. As the focus is not only on instrumental technique, deeply emotional moments can be reached in the process. This was reported by a male guitarist as follows:

“I like to remind players in an ensemble that we are actually there to seek something more than a “correct” reading of a piece of repertoire, that there are other things available emotionally, spiritually or, however, you want to describe experiences of near-transcendent states.”

Such insight brings together the affective and the collaborative dimension of music making. Learning together involves an empathic, emotional, kind of musical intention, where skills can be developed within the group. This process can, in turn, help and sustain the ongoing dynamics of the lesson. Along these lines, a male pianist teacher added that:

“In the collective classes, [the] more the group is close-knit, the more the expressivity increases. And this usually corresponds to the number of days they play together.”

As affectivity^1^ has an impact on what skills are acquired and optimized, so do the histories of interaction among the group: how well they know each other, how long they have been playing together, etc. According to a female organist, this may be linked to another skill, the individual’s listening ability:

“Instrumental technique is important, but in collective classes you need to focus also on the “listening ability” of the student, in a way that they learn to go with the group.”

Ensemble skills like empathy, affectivity, and listening, are certainly not only acquired within a group. While learning music together may help them to be optimized collectively, the influence from a broader cultural and historical environment is also present. This interplay between groups and broader ecological constraints may create novel relationships and interactivities even in contexts that are not explicitly collaborative (see Høffding and Satne, 2019). In the following excerpt, for instance, a female pianist highlighted the need for collaborative learning even in one-to-one settings:

“When my students are playing individually, then they sometimes need me to participate in the music they are playing. I can emphasize certain gestures to help them be more expressive. I would not say that I specifically teach students to do a certain gesture, no. Sometimes, if they have a stiff pose, then we can practice how to start a piece together. You need a beginning gesture. It is an *ad hoc* way to add something with a student who is already at a good level. I don’t really plan these things, though. Instead, we can work together on hand gestures, and breathing. Sometimes I show them how a conductor emphasizes certain gestures to have everyone in the orchestra play together, sometimes it also helps me. I could use this myself too. It is like you cannot develop an expressive style on your own. Rather you need to be immersed in a community and get inspiration from others.”

When peers are not present, the teacher may also be involved – and participate more directly – in the student’s music making. This can contribute to keeping the lesson interesting and under control. As reported by a male violinist:

“Knowing how to create a good class environment; that is fundamental to develop a good teacher-student relationship. If the class displays a good atmosphere (safe and reassuring), then the students come happily to class and feel very motivated.”

As musical skills are always fundamentally immersed in the learner’s cultural identity, affective states and capacity to listen and empathize with others (which helps maintain a safe learning environment) can provide an optimal base from which relationships, activities, and musical skills emerge and develop.

##### Learning skills

The previous quotes show the key role of different ensemble skills for the maintenance and functioning of group settings. In what follows, we focus on those skills that help facilitate the acquisition and mastery of diverse musical actions, ideas, and interactions, leading to enhanced learning experiences and outcomes. A collective approach to music pedagogy poses interesting challenges for both teachers and students. Considerations from our participants regarding what learning skills are acquired as the lesson unfolds may help us better characterize the dynamics of the lesson and its main features. Consider the following two quotes from a male guitarist and a female pianist, respectively:

“There is a certain energy that comes from working and learning in a group. I think students learn a lot from each other as well as from the instructor. There are kinds of music that can only be realized through team effort. This kind of effort is very appealing to me in a fundamental, emotional way. I find goals achieved through ensemble work much more satisfying than goals achieved through individual effort alone. Perhaps humans are “programed” for this?”

“In group class one of the most important aspects is observing and listening to others while being engaged in music, or music-related activities. Cooperation and finding mutual interests in music is more relevant in a group class setting. In group class, students are also able to evaluate their own strengths and weaknesses with regard to the class’ mean.”

Not only do affectivity and listening play an important role in ensuring that the group functions well as a whole, they also help enhance different learning modalities based on cooperation and finding a mutual interest. Interestingly, this involves a self-evaluation of the student’s skills, compared with the average displayed by the group. This is confirmed by another female pianist, who shares the following personal experience:

“I teach the same way to individuals and groups. For the student, however, it changes a lot. Indeed, when a student is with another student, he or she can get much information and can compare himself or herself to the other in ways that can result in a better understanding of a particular technical issue. When a student is alone with me, he or she can only get something from me. But when other students are there, this makes the whole learning process richer. For example, you can see how the others face a given musical problem, and then you can try to relate this to your own experience. It is useful because you are really at the same level of the others. What are your limits? This is a way to learn them.”

Being exposed to others and immersed in the collective setting of the lesson may indeed be considered as an important learning resource. The following comment, from the same respondent, captures the spirit in which the previous insights are offered:

“If I want to show what kind of expressive gesture you need for a piece, I teach this probably in the same way in individual or collective classes. What changes, however, is how the class responds and how expressivity emerges as a unified gesture that everyone contributes to create. The students certainly can be supportive and comment on each other, but usually they do not instruct their colleagues from a technical point of view. But this probably depends on the fact that I have been teaching only professional pianists – maybe amateurs would behave differently and participate more actively. With professionals it is a different story. In my own experience when I was a student myself and we had group sessions, it was part of the lesson to give feedback to our colleagues. But as a teacher, I do not do that. It was okay for me as a student, but I can understand that people might be shy and do not want to give and receive critiques. However, things may change when you start to know your colleagues better.”

The learners’ similar level of expertise may help them empathize with each other and face a range of musical challenges collectively. Such a “problem solving” approach, however, can be easily accompanied by “problem-finding” solutions. Here, the worry to *solve* a given musical problem is traded for a free musical exploration, which may still provide important benefits to each other’s music making. Students can directly observe and listen to their peers, and this can stimulate the group members to challenge themselves and, in turn, help each other. From the viewpoint of a male drummer:

“[A teacher’s satisfaction lies in] the fact that it can trigger a healthy competition between participants. Dealing with different levels it is also a joy whether a skilled student may be encouraged to take a “teaching” role for his/her fellows. This would encourage both, strengthen also the acquired […] knowledge.”

A similar learning skill was highlighted by a male guitarist, who also indicated the role of responsibility and open dialogue in two distinct quotes:

“Students have to take more responsibility for the role they play in the group and the effects their actions or inaction may have on the collective. In a large group, students can learn from each other as well as from the instructor.”

“When everyone is working well together in an ensemble, there is a feeling of exhilaration and “flow” coupled with a kind of edginess that comes from the multiplied potential for error or problems. In a classroom setting, where discussions are taking place, a focused group is capable of developing greater insight and depth because of the diversity of experience and perspective. That feeling of being part of a group discovery or special collective insight can be quite magical.”

According to the respondents, collective learning can help students develop their musical abilities in a variety of ways, including becoming more responsible for their own learning, comparing skills across the group, and generating collective insights.

#### Discussion

When looking at *Ensemble skills*, “affectivity”, “listening”, and “empathy” emerged as key aspects of collective learning. Particularly, they are thought to ensure a positive experience in group settings, facilitating dialogue and wellbeing. Keeping the group interested and within a safe environment is indeed no easy task, and strategies to maintain the lesson as pleasant and appealing can thus play an important role. This can favor the emergence of *Learning skills* such as “comparing yourself to the class” and the “development of responsible ways of learning,” When the group feels safe, mutual learning can unfold as students make meaningful comparisons between themselves and engage in constructive exchanges, leading to what one teacher defines as “healthy competition.” By doing so, students not only become more aware of challenges and possibilities to enhance their learning further but also take more responsibility: they participate more directly in the unfolding dynamics of the lesson, taking up a “teaching role” if needed (Schiavio et al., 2018). Developed from an educator’s perspective, such insights may offer a richer comprehension of the dynamics of teaching music collectively. In particular, these findings point to an understanding of learning as a holistic experience – one that involves experiential dimensions that go well beyond the mastery of motor sequences (Borgo, 2005, 2007).

Consider for example how affectivity is involved in the maintenance of a stable group dynamic. The joy of interacting spontaneously and the exchange of mutual opinions and insights about a given musical challenge is an important resource to emphasize the emotional power of music making (see Juslin and Laukka, 2003; van der Schyff and Schiavio, 2017). As cross-disciplinary research in music increasingly highlights, the deep connection between action, music, and emotion lies at the basis of music cognition (see e.g., Cochrane et al., 2013; Krueger, 2015, 2019; Schiavio et al., 2017), and reinforcing such connection through collective learning can provide an important ground for achieving meaningful musical outcomes. Of course, emotional engagement does not only involve active forms of music making. Instead, the ability to listen to peers, and make sense of the various forms of musical participation within the group, allows the learner to develop multiple series of sensorimotor activities with specific emotional connotations, leading to positive experiences where everyone seems comfortable. A number of contributions point to this intimate connection, recognizing the importance of the bidirectional dependency between being together emotionally and sharing a musical experience (see e.g., Overy and Molnar-Szakacs, 2009; Burnard and Dragovic, 2014a, b; Schiavio et al., 2019b). This is best understood when considering the notion of empathy, a state that indicates the “ability to understand or feel the experience of another person” van der Schyff and Krueger (2019). The teachers in this study thus recognize empathy as an important driver for establishing a meaningful learning environment, one where students can adapt to others, developing strategies to optimize such interaction (see Silverman, 2014). This mechanism is probably facilitated by the similar expertise of the learners in situations of collective teaching. Note for instance how the respondents mention their students’ capacity to self-evaluate in light of what the others are doing. This may have positive effects on the student’s psychological states, particularly as “perfectionism” is increasingly understood as a negative influence on the musicians’ wellbeing (see Araújo et al., 2017). By realizing that others face similar challenges, and display their own limits, collective music making can mitigate the drive to perfectionism. In a similar vein, learning from each other and sharing responsibilities for their own learning, may alleviate one’s desire to feel “perfect”. In what follows, we provide additional grounding to the insights developed here by looking at a dataset retrieved from teachers working at the Royal College of Music.

## Study 2

### Methods

#### Participants

The respondents were selected from an online survey investigating conservatoire students’ and teachers’ views on a variety of musical performance skills. In total, 19 respondents (12 women) were selected for the purpose of this study (age: *M* = 49.26 years, *SD* = 12.96 years, range: 35–74 years). All respondents were teachers at the Royal College of Music, where they teach in a variety of settings such as one-to-one lessons, chamber coaching, orchestral sectionals and masterclasses, as well as being active performers too. Four respondents performed exclusively as soloists, six performed both as soloists and within small or large ensembles, and nine performed exclusively in small or large ensembles. Most of the respondents played piano (*n* = 6), followed by cello (*n* = 3), violin (*n* = 2), recorder (*n* = 2), percussion (*n* = 2), flute (*n* = 1), harpsichord (*n* = 1), organ (*n* = 1), and trombone (*n* = 1). Western classical music was their only performance genre.

#### Materials and Procedure

The study protocol was approved by the Conservatoires UK Research Ethics Committee (CUK REC), and all respondents gave their informed consent prior to taking part in the study. A personal invitation to take part in an online survey was sent to conservatoires, academies and music schools worldwide. Apart from demographic information (age, nationality, and sex), musical background information (e.g., principal instrument, starting age of principal instrument) and current musical activities, the main part of the survey contained questions about the perception of the skills needed to pursue a career as a music performer^2^. The types of skills presented included (a) artistic (musical) skills, (b) technical (instrumental) skills, (c) ensemble skills, (d) learning (practice) skills, (e) presentation (stage) skills, (f) career skills, and (g) life skills (related to performance) (see Williamon et al., 2017, for discussion). In the final section of the survey, participants were asked to describe specific skills that typically fall under each of the seven categories. It was noted that these skills may be particular to the respondent or to their instrument, or they may be skills that are common to all musicians. The same skills could be listed more than once under different categories, if appropriate.

#### Data Analysis

A deductive thematic analysis was utilized to discover overarching themes in the two open responses related to the final section of the survey: *Ensemble skills* and *Learning skills*. This approach consisted of the following five phases: familiarizing, generating initial codes, searching for themes, reviewing themes, and defining and naming themes. The first phase involved reading the responses several times to familiarize oneself with the data set and note down first ideas. In the second phase, codes of interest were generated and applied to the whole data set in a systematic fashion, before several codes were combined into themes in the third phase. Subsequently, these themes were reviewed and, if necessary, refined in phases four and five, respectively. As a last step, compelling and illustrative quotes were selected and integrated into the report.

#### Results

##### Ensemble skills

The most frequently reported ability or skill describing collaborative forms of music making was “listening.” Although it was mentioned by 13 out of 19 respondents, the associations and implications of listening skills differed from performer to performer. Some highlighted “listening” as part of what seems – *prima facie* – a very linear process involving listening (perception) and responding (action) to other musicians or instruments. This juxtaposition of action and perception as distinct moments could lead one to believe that listening is conceived of as a passive process whereas responding is active. In fact, the responses imply that musicians conceptualized “listening” as an active process. For example, a male cellist emphasized the importance of “listening carefully” for successful musical engagements, while a female violinist stressed “the ability to listen acutely at all times” during practice and performance. In both cases “listening” is not reducible to a passive behavior, one where an external stimulus is perceived and coded subconsciously. Instead, “listening,” in this context, arguably requires an active engagement with the surrounding environment. This resonates with literature that emphasizes the tight coupling of perception and action in musical experience (see Maes et al., 2014; Novembre and Keller, 2014). As such, “listening” can be here understood as part of a more complex process where attention, emotion, awareness, and physical effort form a structural unity. Listening allows cohesion of the group and consistency in performance and learning, and can lead to enhanced musical outcomes and experiences. Listening skills thus play an important role in different ensemble settings and may require different subskills depending on the context. For instance, a female flutist described listening skills for duets and trios as follows.

“Again, these are skills I use on students from a very early start. Playing duets using simple notes/rhythms encourages pupils to listen and hear where the music fits in. Canons work very well in making a student count as the single line means the music sounds the same even when 2/4 bars part. Trios mean more listening-learning who starts a piece – how to play together without a conductor. Following the part music on one page (e.g., three parts on one page) is really useful visually and aurally and helps students bring the piece together.”

Not only does “listening” refer to an active process that involves action, it also appears to be part of a cognitive ability to which several respondents referred to directly or indirectly as “awareness.” This may include a focus on the musical sounds, on the music makers themselves, on the other ensemble musicians, and on the interactions between performers. Again, this should not be intended as an abstract faculty discontinuous from the concrete dynamics of music making. Instead, as a female harpsichordist described listening, it allows one to develop novel “insight into other instrumentalists’ musical aims and problems.” “Listening,” as we saw, is more than “perceiving.” But in a sense, it is also more than an individual property, as it always involves a relationality whereby one can successfully interact with others and engage in musical activities in various ways.

This recalls one of the main themes emerging in study 1: that is, the notion of “empathic skills.” Knowing what intentions and feelings are being developed and enacted in music making seems to be pivotal to working together in an ensemble (see Keller, 2008, 2014). The ability to communicate and cooperate with co-performers during learning and performance emerged as a central topic in our participants’ responses and can be thus seen as fundamental to the success of an ensemble. Importantly, one can have different roles within a team and may need to adapt one’s behavior depending on the social, cultural, and contextual contingencies in which the interaction takes place. Several respondents mentioned the ability to follow and lead as crucial skills for achieving the highest musical standards within an ensemble. For instance, “following” might involve “subverting your own musical expectations to help an ensemble work musically,” as a male organist noted. The ability to subordinate one’s own musical aims to those of the ensemble is also emphasized by a female recorder player, who stressed the importance of “flexibility as a player and person.” Here, the boundaries between performative abilities and empathic skills become blurred, as the role of each performer is understood as fluid rather than fixed or predetermined. The “ability to blend, ability to follow, ability to lead,” for example, is mentioned by a female trombonist as a set of social skills that are constantly negotiated during musical interaction, highlighting once again the transformative experience central to collaborative music-making. In this sense, categories such as learning and performing, as well as listening and acting, become hardly distinguishable.

Indeed, social skills based on such sets of actions were reported to be vital during rehearsals, when musical goals need to be negotiated, adjusted, or re-defined. However, they also play an important role during performance, for instance in the form of non-verbal communication skills. In this instance, communication refers not only to the exchange of information among co-performers, but it also involves the audience – for example, how expressiveness can be perceived by an audience. Some of the main skills necessary to excel in ensemble performances without forgetting the audience are reported by a female pianist who is active both as a soloist and in small ensembles:

“Be expressive in your playing and don’t hide behind others. Communicate with your audience and communicate with other players; have highly developed ability to listen and respond to what you hear.”

This quote illustrates the wide range of fundamental skills such as communication and listening, which are not only relevant in ensemble settings but in many different musical contexts, whether they involve rehearsing or performing. Learning skills constitute a similarly basic set of skills that are applied in various musical settings and contain a number of functionally different subskills, as the following section illustrates.

##### Learning skills

The previous section provides important examples of the complex dynamics involving collaborative music making, performance, and learning. In what follows, we report the range of perceptions and experiences the participants associate with learning music in different settings. A first important aspect is the nature of learning itself, which is understood as a never-ending process. Indeed, learning skills are developed and improved throughout a musician’s career or, as a male cellist put it, “[learning] skills can’t ever be good enough.” This quote also implies that learning skills may change over the course of a musician’s career. Whereas at the beginning of a professional career, the main focus might still be on acquiring technical and expressive skills, dealing with an increasing number of teaching and performance engagements at a later stage requires a different approach to learning and rehearsing. It is fitting, then, that a central theme emerging from our sample’s responses is “time management.” Indeed, time – or rather the lack thereof – guides a musician’s practice schedule and relates to a number of components of successful learning strategies. Given these time constraints, the respondents generally agree that problem-oriented approaches to practicing and learning are most promising. The responses in our online survey show that efficient and effective practice sessions are being perceived as vital components of learning skills. One female pianist emphasized the following abilities necessary for efficient and structured rehearsal:

“Be able to concentrate, be able to structure your practice, be able to prioritize, be able to analyze and know what is important; have highly developed listening skills.”

Another female pianist highlighted the role of structured learning in practice sessions by listing the following tasks:

“How to organize self in a structured way. Develop a bunch of ways to tackle individual elements of technique. Forward planning. A structured plan to prepare for performance involving trial performances, etc.”

Furthermore, findings that quality is more important than quantity were reflected in many responses, emphasizing again the time constraints requiring a musician to learn new material or master technically challenging passages (see Williamon and Valentine, 2000). A male percussionist commented on this skill, which is important in both individual and collective teaching settings. He said:

“Absorbing music quickly, working out the spatial properties of a hybrid multi-instrument set-up, experimentation.”

This excerpt includes aspects that are specific to the instrument being learned (i.e., percussion), but that could, at the same time, be generalized. Consider how different spatial set-ups can also play an important role in learning a piece for violin (e.g., the room reverb, or the response of the instrument to a particularly humid environment). Thinking about the demands of an instrument, which may result in very different practice schedules, a female violinist further reported:

“To be able to practice effectively and at times extremely quickly; to be physically aware in order to meet the demands of one’s instrument.”

Awareness of being physically situated in a specific niche can lead to different options for learning, whereby one has to adjust constantly to specific settings and modify practice accordingly. While developing learning skills necessarily entails playing one’s own instrument, learning may sometimes also take place away from the instrument: it can include a focus on the environment, as we saw, or what one female pianist called “head-practice.” Her account of “learning” aptly summarizes the different facets involved in learning skills when she stated:

“Disciplined and structured practice pattern, including technical warm-up; clear aim within the practice session; structured pattern of learning, including fingering, slow practice, repetition where helpful, […] recording from an early stage in learning new repertoire; structure for practice at home and on days of performance and when traveling.”

To account for both dimensions (learning with and without the instrument), musicians need to be equipped, as a male pianist indicated, with a considerable amount of “self-discipline, self-motivation, [and] determination.” While these personality traits seem to be especially vital in individual teaching contexts, collective tuition may facilitate motivational aspects of learning. Within a group setting, processes of social cognition can boost determination (e.g., feelings of “I don’t want to be the one obstructing progress of the ensemble”), or social contagion can enhance one’s own motivation by observing the motivation of others.

#### Discussion

In this study, we focused on ensemble and learning skills in order to understand what a sample of highly trained musicians, who are active both as performers and teachers, associate with these skills. Our findings suggest that the most important component of ensemble skills is the ability to listen and respond to others. This *active* process of listening and responding takes place in different contexts. For instance, it is part of the rehearsal process where musical goals are articulated and negotiated with other members of an ensemble. One needs to listen to the musical ideas and thoughts of others, compare those with one’s own set of musical beliefs and intentions, and then respond by agreeing or providing suggestions for alternative approaches. Such negotiations may involve both talking as well as playing to demonstrate a musical idea. Likewise, listening and responding is pivotal during performance. Here, only non-verbal cues can be utilized to communicate with and respond to co-performers. These can be bodily gestures, facial reactions and, of course, the musical sound which allows a performer to convey expressiveness and respond musically to his/her co-performers’ playing.

The success of an ensemble can be likened to other professional areas (e.g., industry, politics) where an effective performance necessitates the collaboration of two or more people to achieve a common goal. Working together as a team was thus the second broad theme to emerge from our data. Team work involves the ability to communicate clearly and effectively, while being aware of one’s own role within a group which may change depending on the context. As a leader, one may need to show guidance, conviction, persistence and be able to persuade without neglecting the thoughts and feelings of other ensemble members. As a follower, one needs to listen carefully and blend in with the others to achieve a common musical goal. Although not mentioned explicitly by the respondents, underlying concepts such as trust and empathy seem to be vital ingredients for a successful ensemble performance (see Novembre et al., 2019).

“Time management” emerged as the most important facet of learning skills. Given our sample of RCM teachers, it is perhaps not surprising that components of learning skills are almost exclusively considered through the raster of tightly scheduled teaching and performing diaries. Refinements such as “goal-oriented, structured approaches to learning” thus need to be evaluated with regards to the overarching time constraints articulated by the participants. The importance of focusing on specific learning goals is in accordance with previous literature (Locke et al., 1981) and requires a set of meta-cognitive skills (Concina, 2019). A musician needs to execute and monitor the success of a rehearsal plan, while planning ahead and flexibly adjusting learning strategies. Although 15 out of 19 participants in our study performed in small or large ensembles and all of them teach in different settings (one-to-one, ensemble, etc.), learning skills are framed primarily from an individual tuition perspective. The respondents’ focus on self-discipline and self-motivation does not consider the specific social contexts of ensemble rehearsals where discipline and motivation need to be distributed and shared among all members of a group. It is interesting to note that the collaborative aspects of music making seemingly play a subordinate role – at least, those aspects were not mentioned explicitly when asked to provide specific skills that typically fall under the category of *Learning skills*.

## General Discussion and Conclusion

This article brings together qualitative data on *Ensemble skills* and *Learning skills* from two different groups: music teachers involved in various pedagogical and performative contexts with different skill levels, and music teachers from a conservatoire. While there are intrinsic differences concerning the general goals of their teaching, as well as the educational system in which they operate, our analysis shows a number of overlapping themes.

First, when it comes to *Ensemble skills*, both studies emphasize the ability to listen to each other as a fundamental aspect of joint music making. In both performance and learning settings, being open to one another, and engaging collectively in making music will lead to valuable outcomes regardless of expertise level or genre. This aligns with recent views on the sensorimotor nature of listening experience put forward by Froese and González-Grandón (2019). They propose that sensorimotor expertise is partly guided by audition, as it enables motor adaptations to sonic events as well as anticipatory auditory imagery. In a sense, “listening” (to each other, to the music being generated, etc.) can contribute to successful learning and performing not only because it creates a stream of communication across peers, or between teachers and students, but also because – at a more grounded level – it provides an opportunity for action (see Clarke, 2005; Reybrouck, 2005; Schiavio et al., 2015). Such actions-as-perceptions, therefore, include affective and empathic elements as they bring together a full range of experiences involving perceptual, bodily, and interactive dimensions (Loaiza, 2016; Schiavio and De Jaegher, 2017). They can also transform a solitary activity into a collective effort where musical meanings are negotiated and fluidly integrated into one’s performance (Høffding and Satne, 2019), and can lead to richer understandings of the different roles to be taken and exchanged in a musical passage (see Schiavio and Høffding, 2015). Accordingly, it comes as no surprise that groups with different levels of expertise can benefit from “listening,” conceptualized as an integral part of learning and performing music.

Such insights can also illustrate the main findings emerging from our analysis of *Learning skills*: being receptive to peers and teachers can lead to concrete changes in one’s learning strategies, favoring solutions such as “comparing yourself to the class,” “development of responsible ways of learning,” or putting a major emphasis on “time management.” It is interesting to observe how teachers dealing with a rich variety of pedagogical settings share a mutual agreement concerning their students’ learning. In both collective and individual tuition, open dialogue and reflection can stimulate the musician and the pupil rethink their approach to learning. This resonates with recent accounts on skill acquisition that emphasize the value of collectivity for developing different abilities in sport and music (see e.g., Davids et al., 2013; Schiavio et al., 2019a). Rather than understanding skills as properties of the lone individual, this research suggests that we may in fact consider how external contingencies take an important, even co-constitutive role in skill development, as we are always immersed in a community of practice, with peers, teachers, technology, as well as their associated social and historical dimensions. This research is also concerned with the continuity between individual and collective dimensions of learning, a dimension that is emphasized by the participants of our study. Consider for example how our findings point to the integration of individual goals and collective dynamics in *Learning skills*: in study 1 the teachers recognized the importance of a safe learning environment for learning, where students are free to express themselves and engage in constructive peer-learning and self-assessment (see van der Schyff, 2019). In study 2, while most focused on individual practice, the respondents emphasized how different external constraints (e.g., spatial settings) need to be considered for optimizing practice, as well as self-evaluation and the ability to listen to oneself. The latter, as we discussed, is also the main overlapping theme emerging from our analysis of *Ensemble skills*. This reciprocity between listening, acting, interacting, and exploring the environment as well as the self, reveals the limitations of the standard idea of learning portrayed as a ladder, where skills develop through a linear process of acquisition of external information, individual internal elaboration, and repetition of behavioral outcomes. In fact, it is within the complex, non-linear, interplay of factors internal and external to one’s skillset that group dynamics can be successfully regulated and learning can take place (see also Schiavio and van der Schyff, 2018). Consider how one can develop a particular fingering solution that is easier when practicing alone but can then be re-explored and modified to account for a particular expressive necessity that only emerges when playing with others, leading to online adaptive regulations, or how a phrasing choice can be revisited to convey a better sense of tension, which may be further developed via open discussion with peers and teachers. “Listening” (to themselves and to others) becomes then an enabling condition for meaningful music making, a tool for sonic discoveries that is negotiated and transformed collectively to serve a variety of creative functions (see also van der Schyff et al., 2018; Gutierrez, 2019). Students and performers need to be attuned to their peers, be aware of the environment in which they perform and learn, and adapt themselves to what such a setting may offer. Accordingly, “listening” needs to be understood as a process that is continuous with our capacity to act and engage with others in a musically meaningful task.

Similar considerations are being increasingly discussed among many music scholars interested in creative teaching and learning (see e.g., Haddon and Burnard, 2016) and, at a closer look, resonate with the embodied approach briefly mentioned above. This framework, generally speaking, holds that mental activity is co-constituted by the body and its capacity to act in the world in which it is situated (Thompson, 2007; Chemero, 2009). This means that our capacities to think, communicate, generate meaning, or make music, are co-determined by action-oriented sensorimotor processes involving a whole brain-body system. This provides a step forward from the main psychological approaches developed during the last century to explore human cognition: because mentality cannot be reduced to the analysis of neural patterns, this framework goes beyond classic “neurocentric” perspectives inspired by classic “identity” theories (see e.g., Place, 1956). Similarly, exploring cognition mainly in terms of computations and representations – as in classic cognitivism (see e.g., Pylyshyn, 1986) – would be equally insufficient to capture the full complexity of mind: generalizations about cognitive architectures and their properties can work only if we posit an intrinsic discontinuity between the “hardware” of the flesh and the “software” of the mind. Many scholars, however, warn us that similar positions may dangerously flirt with a Cartesian view that separates *de facto* an abstract, program-level self from the physical facts of our body (see Thompson, 2007; Fuchs, 2017). Importantly, resisting neurocentric theories and classic cognitivism does not make embodied cognition a behaviorist doctrine. Cognition is not simply equated with behavior, but rather entails an equal recognition of internal and external factors. That is, bodily, experiential, emotional, and behavioral activities always take place in a meaningfully rich environment. We are situated in a niche that offers concrete possibilities for action and affords different social and cultural meanings (Chemero, 2009). As the framework of embodied cognition recognizes the profound continuity between physiology, actions, emotion, and the world, it provides an apt counterpoint to the previously considered theories: contrary to neurocentric, cognitivist, and behaviorist doctrines, research in embodied cognitive science also aims to address the subjective, unique, bodily, and emotionally rich experience that living systems develop in a given context (Colombetti, 2014).

Before concluding, it should be noted that bringing together the two studies presented here entails necessary compromises and methodological challenges. As reported, study 1 deals with teachers whose experience reflects a rich variety of educational contexts. By contrast, study 2 describes views from teachers working in a traditional conservatoire focusing on Western classical music. While we deliberately selected groups of participants with different teaching experiences to address the breadth of educational settings, we noted^3^ that participants from study 1 drew out many more ensemble skills and learning skills when compared with respondents from the Royal College of Music. This may be due to differences in data collection, but also to the wider range of student levels experienced by teachers in study 1. As such, more research is needed to look further into the teaching dynamics associated with a particular skill level, genre, style, and culture. This can shed new light on more specific issues and skills that develop from a given context, helping educators reflect on problems with which they can more strongly resonate, and leading to innovative pedagogical solutions systematically tailored to the musical context in which they are situated.

## Data Availability Statement

The datasets generated for this study are available on request to the corresponding author.

## Ethics Statement

The studies involving human participants were reviewed and approved by the Ethics Committee of the University of Graz (study 1) and the Conservatoires UK Research Ethics Committee (study 2). The participants provided their written informed consent to take part in this research.

## Author Contributions

AS and MK developed the rationale of the research and wrote the manuscript. AS re-analyzed data from study 1. MK re-analyzed data from study 2. AW provided comments and suggestions that were implemented in the final draft. All authors approved the final version of the manuscript.

## Conflict of Interest

The authors declare that the research was conducted in the absence of any commercial or financial relationships that could be construed as a potential conflict of interest.

## Footnotes

1 With this term, we refer to our capacity to feel emotions.

2 In this sense, the present study was designed to elicit more specific answers than those emerging in study 1.

3 We thank one reviewer for pointing this out.
